# Bioinformatic Analysis of Oxalate-Degrading Enzymes in Probiotics: A Systematic Genome-Scale and Structural Survey

**DOI:** 10.3390/microorganisms13112553

**Published:** 2025-11-08

**Authors:** Shengda Du, Ke Sun, Bo Xiao, Zhihua Liu

**Affiliations:** 1 College of Life Sciences, Northwest A&F University, Yangling 712100, China; 18790597018@163.com; 2 Institute for Immunology, School of Basic Medical Sciences, Tsinghua University, Beijing 100084, China; sunk22@mails.tsinghua.edu.cn; 3 Department of Urology, Research Center for Urinary Disease, School of Clinical Medicine, Beijing Tsinghua Changgung Hospital, Tsinghua University, Beijing 102218, China; 4 Tsinghua-Peking Center for Life Sciences, Tsinghua University, Beijing 100084, China

**Keywords:** kidney stone disease, probiotics, enzymes, oxalate degradation, AlphaFold, TM-align

## Abstract

This bioinformatic study provides a comprehensive theoretical assessment of oxalate-degrading enzymes in probiotics. Kidney stone disease is a common urological disorder with rising global incidence, largely driven by the precipitation of insoluble calcium oxalate salts. Current treatments—including thiazides, lithotripsy, or ultrasound fragmentation—often show variable outcomes and high recurrence rates. Here, we systematically assessed the oxalate-degrading potential of 38 probiotic species listed in the List of Cultures Available in Food (China National Health Commission) along with selected next-generation probiotics. Using BLASTp homology searches, we identified seven strains carrying both *oxalyl-CoA decarboxylase* (*OXC*) and *formyl-CoA transferase* (*FRC*) genes, one encoding *oxalate decarboxylase* (*OXDC*), and three harboring subunits of *oxalate oxidoreductase* (*OOR*). Additionally, seven species from international probiotic lists (*EFSA QPS* and *AEProbio*) were analyzed, among which two carry both *OXC* and *FRC* genes. We prioritized strains with the coupled OXC-FRC pathway or OOR enzymes, examined catalytic site conservation by multiple sequence alignment, and performed AlphaFold-based structural prediction with Template Modeling (TM)-align scoring. Species with TM-scores >0.8 exhibited highly conserved folds, suggesting functional oxalate degradation capacity. These findings provide theoretical guidance for identifying probiotic candidates with oxalate-degrading activity and establish a framework for developing next-generation functional probiotics to alleviate kidney stone disease.

## 1. Introduction

Urolithiasis, commonly referred to as kidney stone disease, is a prevalent urological disorder affecting nearly 10% of the global population [1]. Its incidence is influenced by multiple factors, including diet, geography, water composition, sex, age, and genetic background [2,3,4]. Recurrence rates are high, ranging from 4% to 75% [1], and in high-risk populations, kidney stones often become a lifelong burden [5,6]. In severe cases, complications such as recurrent urinary tract infection, renal impairment, and even kidney failure may occur [7]. Despite advances in surgical and pharmacological management, no universally curative or recurrence-preventing therapy currently exists [8,9].

Current treatments are divided into preventive interventions and surgical procedures. Preventive strategies aim to identify high-risk individuals and modify diet and lifestyle under medical supervision. However, compliance is poor, and long-term implementation requires significant healthcare resources, limiting feasibility in many regions [10,11]. Surgical approaches such as percutaneous nephrolithotomy (PCNL) and retrograde intrarenal surgery (RIRS) achieve high clearance rates but still carry risks of complications [12,13]. Extracorporeal shock wave lithotripsy (ESWL) is less invasive but often requires repeated sessions, reducing efficiency and increasing cost [14]. Collectively, these limitations underscore the need for novel, non-invasive strategies. Recent research has increasingly pointed to the gut microbiota as a promising target for such interventions [15,16,17].

Elevated urinary oxalate levels are a major risk factor for calcium oxalate kidney stone formation. The gastrointestinal tract (GIT) plays a central role in systemic oxalate balance because dietary oxalate can be absorbed or metabolized by intestinal microbiota prior to entering systemic circulation. Dysbiosis, reduced abundance of oxalate-degrading bacteria such as *Oxalobacter formigenes*, or increased intestinal oxalate absorption can elevate plasma and urinary oxalate levels, ultimately promoting stone formation [18,19,20]. Therefore, probiotics capable of degrading oxalate in the gut may help regulate systemic oxalate levels and serve as preventive agents against kidney stone disease [21,22,23].

Kidney stones arise primarily from the precipitation of insoluble salts, with calcium oxalate as the dominant component [24,25]. Hyperoxaluria is a major risk factor for calcium oxalate stone formation [26]. Emerging evidence highlights the gut microbiota as a key regulator of systemic oxalate levels [27,28,29]. Several oxalate-degrading bacteria, including *Oxalobacter formigenes*, *Lactobacillus acidophilus*, and *Bifidobacterium animalis* subsp. *lactis*, have been shown to metabolize oxalate through specialized intracellular enzymes [27]. Known oxalate-degrading enzymes include oxalate oxidase (OXO), oxalate decarboxylase (OXDC), oxalyl-CoA decarboxylase (OXC), formyl-CoA transferase (FRC), and oxalate oxidoreductase (OOR).

These enzymes degrade oxalate through distinct biochemical mechanisms. Oxalate oxidase (OXO) and oxalate decarboxylase (OXDC), primarily found in fungi and plants, catalyze distinct reactions: OXO oxidizes oxalate to yield carbon dioxide and hydrogen peroxide, whereas OXDC catalyzes the decarboxylation of oxalate to form carbon dioxide and formate. Both enzymes are Mn^2+^-dependent for catalytic activity [30,31]. In contrast, the bacterial OXC–FRC system constitutes a two-step, CoA-dependent pathway. Formyl-CoA transferase (FRC) first transfers CoA from formyl-CoA to oxalate, generating oxalyl-CoA and formate. Subsequently, oxalyl-CoA decarboxylase (OXC) catalyzes the decarboxylation of oxalyl-CoA to yield formyl-CoA and CO_2_, thereby completing a cyclic process that regenerates the CoA donor [24]. Oxalate oxidoreductase (OOR), identified in certain anaerobic bacteria, catalyzes the oxidation of oxalate with the transfer of electrons to ferredoxin, producing carbon dioxide as the sole carbon product under anaerobic conditions. Collectively, these enzymatic pathways illustrate evolutionarily distinct mechanisms of oxalate catabolism across diverse taxa.

**Figure 1:**
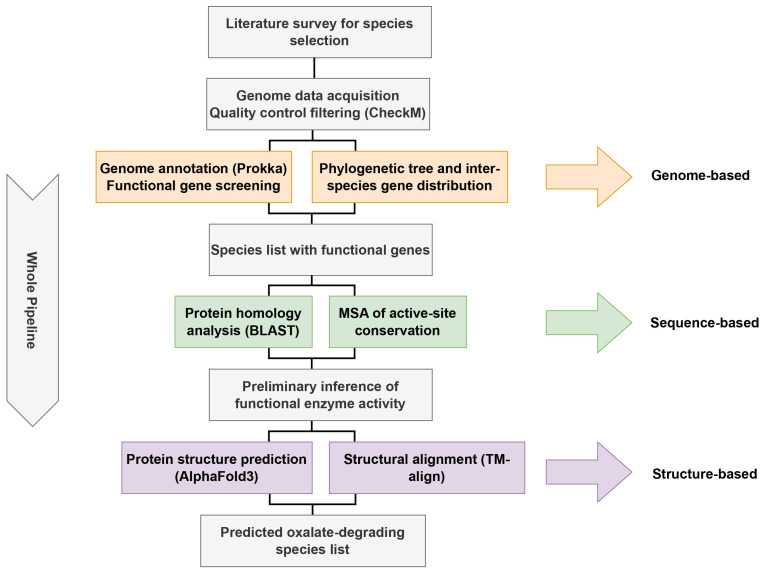
The whole pipeline of this work. Overall workflow of this study, which is organized into three analytical levels: genome-based, sequence-based and structure-based. These levels are distinguished by three different colors in the diagram for better visualization.

However, most studies have focused on a few species or strains, leaving the oxalate-degrading potential of other widely used or next-generation probiotics largely unexplored. This study addresses this gap by systematically screening probiotic genomes for oxalate-degrading enzymes using a three-level pipeline: genomic distribution, sequence conservation, and structural similarity (Figure 1). By combining annotation, multiple sequence alignment, AlphaFold prediction, and TM-align comparison, we identify novel probiotic candidates with potential oxalate-degrading capacity, providing new directions for microbiota-based interventions against kidney stone disease.

## 2. Materials and Methods

### 2.1. Genome Retrieval and Quality Control

We analyzed 38 species listed in the List of Cultures Available in Food (China National Health Commission), together with selected next-generation probiotics (Supplementary Table S1). A total of 715 genomes were retrieved from NCBI (Supplementary Table S1) [32,33]. Genome quality was assessed using CheckM v1.0.12 [34] with thresholds of >95% completeness and <3% contamination. For each species, the best-quality assembly was chosen for analysis.

### 2.2. Gene Annotation and Phylogenetic Analysis

Representative genomes were annotated using Prokka v1.13 [35]. Genes encoding OXO (EC 1.2.3.4), OXC (EC 4.1.1.8), FRC (EC 2.8.3.16), OXDC (EC 4.1.1.2), and OOR (EC 1.2.7.10) were identified. Phylogenetic trees were constructed using GTDB-Tk v2.4.0 [36] and visualized in iTOL [37]. Functional gene distributions were mapped onto the phylogeny.

### 2.3. Protein Sequence Homology and Catalytic Site Conservation Analysis

Homology of protein sequences extracted from the annotated genomes was assessed using BLAST+ v2.13 [38] against Protein Data Bank references [39] (Supplementary Table S3). BLASTp searches were performed with an E-value cutoff of 1 × 10^−7^, retrieving the top hit for each query (–max_target_seqs 1) to ensure statistically significant and biologically relevant matches. *Lactobacillus gasseri* (GCF_000014425.1) served as an internal benchmark for OXC and FRC. For OOR, a reference sequence database (including three subunits) was constructed, and BLASTx searches were conducted against genome annotation files to identify residual subunits. This OOR database used for Blastx are available in a public GitHub repository via https://github.com/ShengdaDu/OOR-database-for-Blastx (accessed on 24 October 2025). Multiple sequence alignments (MSA) were generated with MAFFT via https://www.ebi.ac.uk/jdispatcher/msa/mafft?stype=protein (accessed on 7 August 2025) [40,41] and visualized with Jalview v2.11.4.0 [42]. Literature-curated catalytic residues were mapped to evaluate conservation.

### 2.4. Protein Structure Prediction and Comparison

Predicted structures were obtained via AlphaFold online platform https://alphafoldserver.com/ (accessed on 11 August 2025) [43,44]. Structural similarity was assessed using TM-align, with TM-scores >0.8 considered conserved folds, as TM-scores above 0.8 generally indicate nearly identical global folding and reliable structural alignment [45]. RMSD cutoffs ≤ 2.0 Å were applied to ensure close atomic alignment. Structural visualizations, including per-residue confidence scores (pLDDT) and the results of TM-align superposition were generated with PyMOL [46].

## 3. Results

### 3.1. Quality Control of Genome Data

A total of 715 genomes were retrieved from NCBI for 38 species listed in the List of Cultures Available in Food and selected next-generation probiotics. Of 715 genomes analyzed, 89 high-quality representatives met thresholds. These comprised 32 traditional probiotics (e.g., *Lactobacillus gasseri*, *Bifidobacterium animalis*) and 57 next-generation probiotics (e.g., *Akkermansia muciniphila*, *Blautia hydrogenotrophica*), thus traditional and next-generation probiotics were comprehensively represented in the present study.

### 3.2. Distribution of Oxalate-Degrading Genes Across Species

To investigate the distribution of oxalate-degrading genes across species, we annotated the 89 representative genomes using Prokka. Among traditional probiotics, seven species were found to harbor both *OXC* and *FRC* genes, including *Bifidobacterium animalis, Lactobacillus gasseri*, *Lactobacillus acidophilus, Lactobacillus johnsonii, Lactobacillus helveticus, Lactobacillus kefiranofaciens*, and *Limosilactobacillus reuteri*. Notably, two *FRC* paralogs were detected in *L. helveticus* and *L. kefiranofaciens*. Neither *OXC* nor *FRC* genes were found in next-generation probiotics. Instead, *OOR* was identified in *Blautia marasmi*, *Blautia pseudococcoides*, and *Blautia hydrogenotrophica*; and *oxalate decarboxylase* (*OXDC*) in *Lachnospira eligens*.

**Figure 2:**
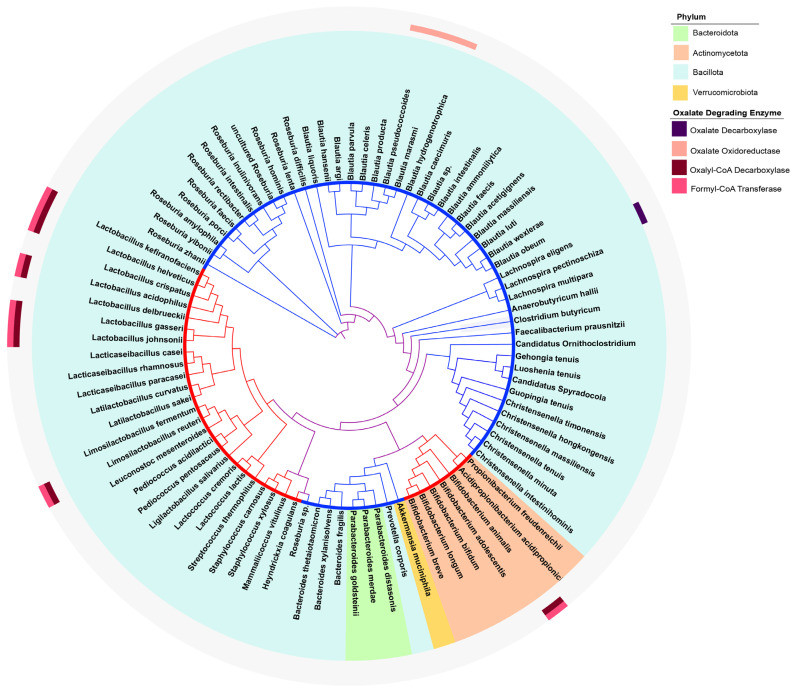
Phylogenetic tree of the selected species and distribution of functional genes. Species from different phyla are shaded with distinct background colors. The presence of enzyme-encoding genes is indicated on the outer ring with colored markers, while gray denotes absence. The inner ring distinguishes probiotic categories, with red representing traditional probiotics and blue representing next-generation probiotics. Branch colors denote clade types: red for traditional probiotics, blue for next-generation probiotics, and purple for clades that phylogenetically comprise both traditional and next-generation probiotics.

To further investigate the phylogenetic distribution of oxalate-degrading genes, we constructed a species phylogeny using GTDB-Tk and overlaid the gene distribution onto it (Figure 2). The analysis showed that *OXC* and *FRC* typically co-occurred and formed a distinct cluster within the genus *Lactobacillus*. Within this cluster, *L. helveticus* and *L. kefiranofaciens,* both carrying two copies of *FRC*, were grouped together. Outside this cluster, *L. reuteri* also carried both *OXC* and *FRC*. In contrast, *OOR* genes were confined to a separate cluster within the *Blautia* lineage.

Taken together, these results indicate that *OXC* and *FRC* consistently co-occur and are largely restricted to traditional probiotics, whereas among next-generation probiotics, only a small subset of species harbor either *OOR* or *OXDC*.

### 3.3. Protein Homology and Active Site Conservation

To assess protein-level conservation of oxalate-degrading genes, we conducted BLAST homology analyses. Two datasets were generated: one using PDB reference sequences as queries (Supplementary Table S4) and the other using *L. gasseri* OXC and FRC proteins as references (Supplementary Table S5).

When compared with PDB references (Supplementary Table S4), OXC proteins displayed sequence identities of ~50%, with the lowest observed in *B. animalis* (48.60%). FRC proteins (excluding strains carrying duplicate copies) showed ~45% identity. Among strains with duplicate FRC copies, no sequence was retrieved for *L. helveticus* FRC_1, while FRC_2 displayed 36.10% identity. In *L. kefiranofaciens*, FRC_1 and FRC_2 exhibited 51.71% and 40.93% identity, respectively.

When comparing to *L. gasseri* (Supplementary Table S5), all OXC and FRC proteins, except *B. animalis* OXC (49.83% identity), showed >70% identity. By contrast, the OXDC protein of *Lachnospira eligens* showed only 34.57% identity. Notably, in the *OOR* gene comparison (Supplementary Table S5), only a single subunit was initially detected per strain, despite the enzyme complex requiring three subunits. Additional BLASTx searches against the complete genomes successfully identified the missing subunits.

Taken together, these results suggest that species harboring *OXC* and *FRC* genes are likely to encode functional enzymes; *Blautia pseudococcoides* and *Blautia hydrogenotrophica* may carry complete *OOR* gene clusters; and *Lachnospira eligens* may potentially exhibit OXDC enzymatic activity.

### 3.4. Multiple Sequence Alignment of Catalytic Sites

**Figure 3:**
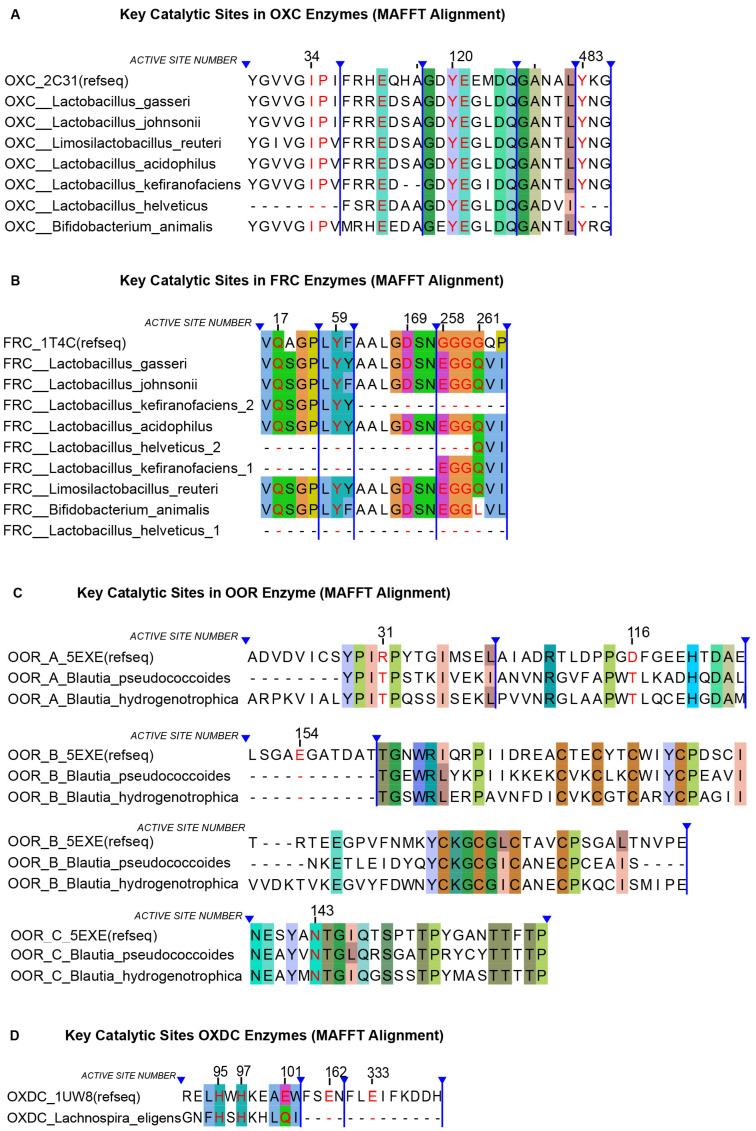
MSA of protein sequences from different species based on reference sequences (MAFFT). Catalytic active sites are highlighted in red font. Background shading indicates sequence conservation according to the ClustalX color scheme in Jalview. Regions marked with blue lines represent hidden sequence segments in the software. The figure is divided into four panels: (**A**) OXC, (**B**) FRC, (**C**) OOR, and (**D**) OXDC.

To assess the conservation of catalytic residues in oxalate-degrading enzymes, MSAs were generated using MAFFT and visualized in Jalview (Figure 3). Among traditional probiotics, OXC protein sequences conserved all annotated catalytic residues except in *L. helveticus*, where the auxiliary binding residues Ile-34 and Pro-35 and the catalytic residue Tyr-483 were absent. For FRC, the catalytic residues Glu-17, Tyr-59, and Asp-169 were highly conserved across species, except in those carrying duplicate FRC genes. At the flexible loop (residues 258–261, GGGGQ), partial conservative substitutions were observed, although the critical residues Gly-259 and Gly-260 remained unchanged. Both FRC paralogs in *L. helveticus* lacked all conserved catalytic residues. Interestingly, in *L. kefiranofaciens*, the two FRC paralogs retained complementary portions of the catalytic region, though Asp-169 was missing in both.

For next-generation probiotics, the OXDC sequence of *Lachnospira eligens* retained auxiliary binding residues His-95 and His-97 but lacked the catalytic residues Glu-162 and Glu-333. In *B. pseudococcoides* and *B. hydrogenotrophica*, both of which carried OOR clusters, subunit A exhibited conserved substitutions at Arg-31α and Asp-116α; subunit B lacked Glu-154γ but preserved downstream binding residues despite being shorter than the reference; and subunit C maintained conservation at Asn-143β.

Overall, these findings suggest that within the OXC–FRC system, *L. helveticus* likely lacks functional FRC activity, whereas other species may retain enzymatic potential, but *L. kefiranofaciens* contains an incomplete *FRC* gene sequence that may indicate residual conservation of key regions. Among next-generation probiotics, *L. eligens* is unlikely to encode a functional OXDC, while *B. pseudococcoides* and *B. hydrogenotrophica* may exhibit functional OOR activity.

### 3.5. Structural Prediction and Alignment

To evaluate the enzymatic potential of candidate functional genes at the protein structure level, we performed structure prediction using AlphaFold and structural superposition analysis with TM-align. Because both *FRC* genes in *L. helveticus* lacked all critical catalytic residues, they were not included from subsequent analysis.

**Figure 4:**
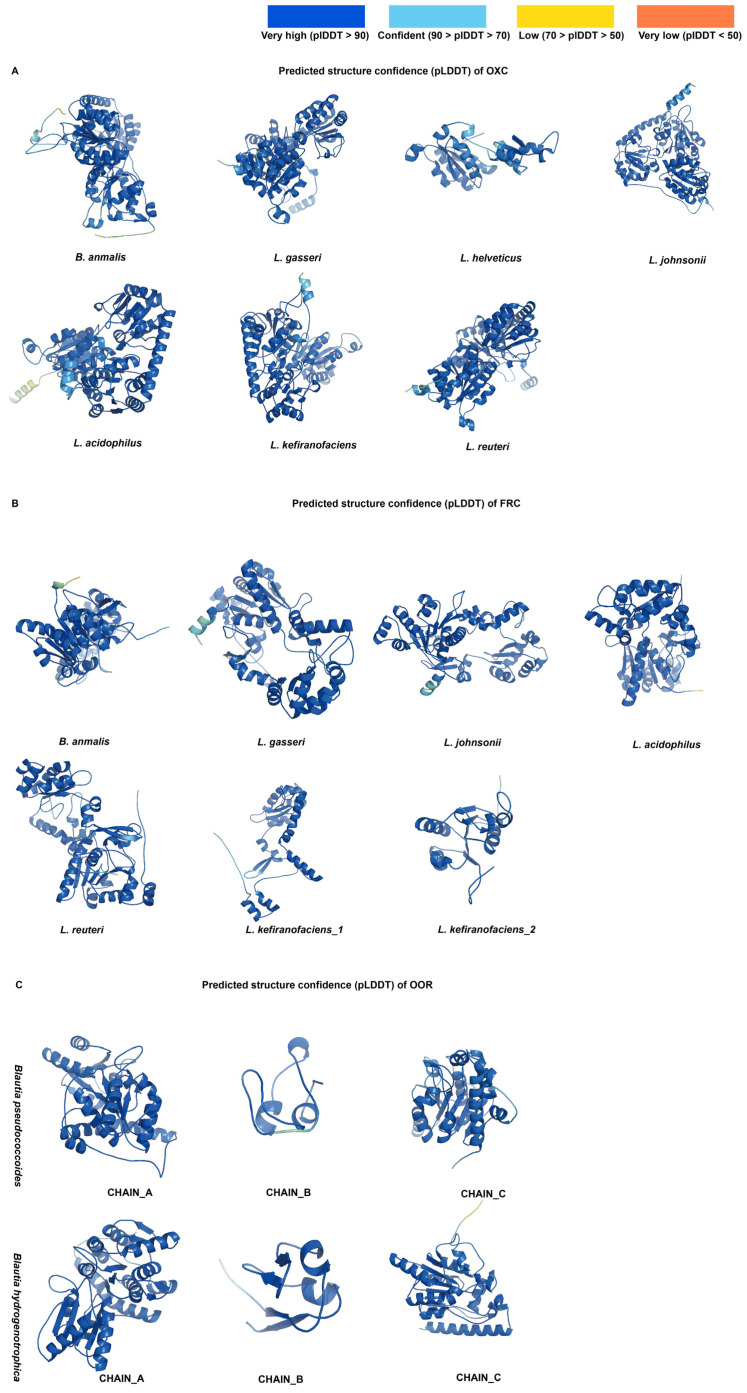
Predicted confidence scores of protein structures generated by AlphaFold. Colors represent the confidence level of structural prediction: dark blue indicates very high confidence (score > 90), light blue indicates confident regions (score 70–90), orange indicates low confidence (score < 70), and red indicates very low confidence (score < 50). The figure consists of three panels: (**A**) OXC, (**B**) FRC, and (**C**) OOR (three subunits from two species).

**Figure 5:**
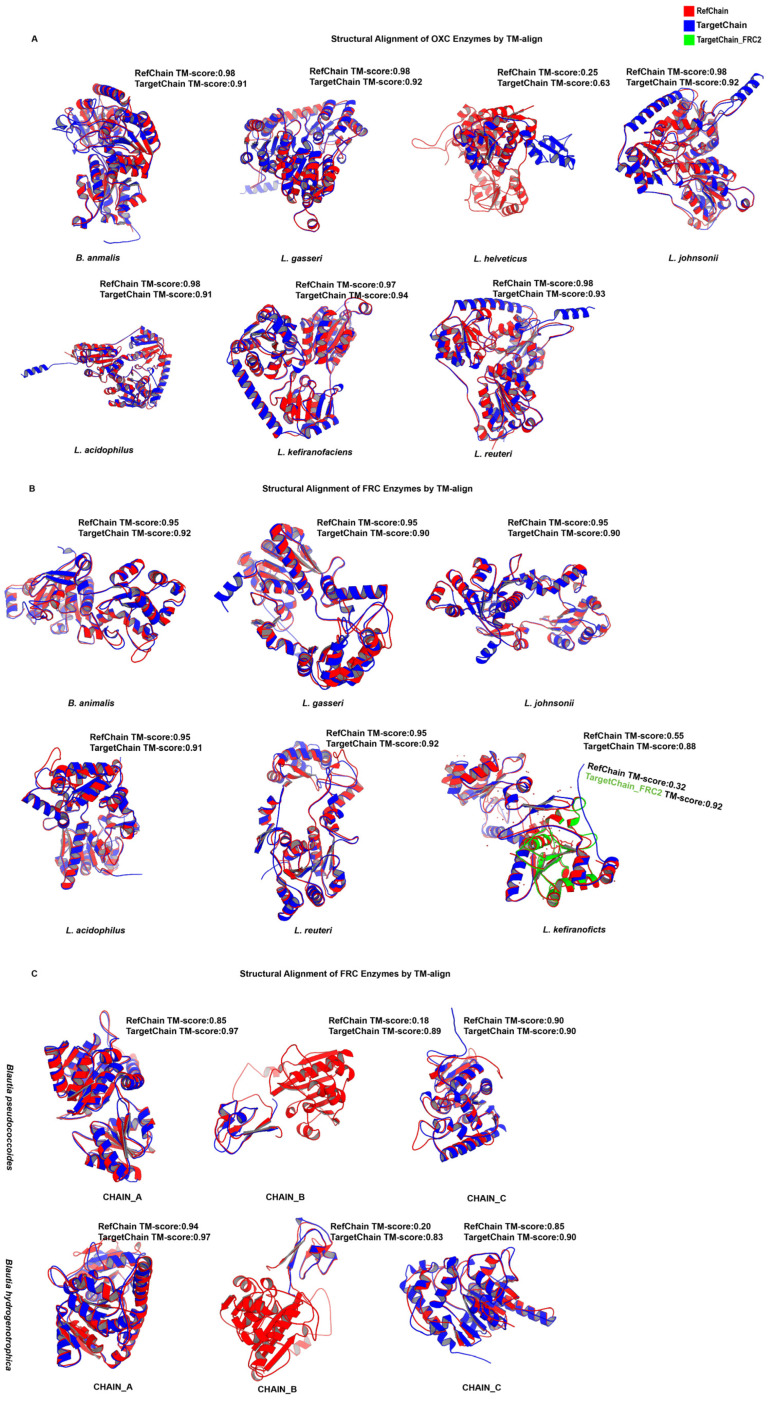
Structural superposition of predicted proteins with reference structures using TM-align. In the overlays, red represents the reference structure, blue represents the query structure (for *L. kefiranofaciens* in Figure 3 this corresponds to FRC_1), and green represents FRC_2. The figure consists of three panels: (**A**) OXC, (**B**) FRC, and (**C**) OOR. Normalized alignment parameters (TM-scores) are indicated in the upper right corner of each panel.

Inspection of the AlphaFold confidence maps (Figure 4) showed that most predicted structures were predominantly colored blue, indicating high confidence, with only a few localized regions in yellow. Structural alignment results further supported these observations. For *OXC*, all species harboring the gene exhibited strong structural overlap with the reference enzyme, except *L. helveticus*, which showed only partial alignment (Figure 5). For FRC, an intriguing case was observed in *L. kefiranofaciens*: when both predicted FRC proteins were aligned with the reference, their structures together displayed a high degree of similarity. The remaining five species carrying *FRC* also showed strong alignment with the reference. For *OOR, B. pseudococcoides* and *B. hydrogenotrophica* displayed consistent alignment patterns across the three subunits: subunits A and C aligned well with the reference, whereas subunit B overlapped strongly only in part of the structure.

Quantitative TM-align parameters corroborated these findings. For OXC, all species except *L. helveticus* had TM-scores above 0.9 and RMSD values below 2 Å, exceeding the commonly accepted threshold (TM-score > 0.8). For FRC, the two copies in *L. kefiranofaciens* produced asymmetric results, with one TM-score below 0.8 and the other above; by contrast, the other five species consistently scored >0.8 with RMSD < 2 Å. For OOR, subunit A of both *B. pseudococcoides* and *B. hydrogenotrophica* achieved TM-scores >0.8, subunit B showed low reference scores (<0.3) but high target alignment (>0.8), and subunit C consistently scored above 0.8.

Together, these results align with our catalytic-site analyses and indicate that the OXC and FRC proteins in *B. animalis*, *L. gasseri*, *L. acidophilus*, *L. johnsonii* and *L. reuteri* are structurally equivalent to their reference counterparts. In *L. kefiranofaciens*, two FRC-like proteins were identified, each matching complementary regions of the reference sequence. Their combined alignment closely resembled the reference structure, suggesting possible structural complementation that warrants experimental confirmation, while *B. pseudococcoides* and *B. hydrogenotrophica* likely retain OOR activity through highly conserved A- and C-subunits.

In summary, analysis of high-quality representative genomes identified eight candidate probiotic species with potential oxalate-degrading capacity. These fall into two groups with distinct metabolic strategies: (i) six traditional probiotics, including five *Lactobacillus* species and *L. reuteri*, which appear to rely on the canonical anaerobic OXC–FRC pathway; and (ii) the next-generation probiotics *B. pseudococcoides* and *B. hydrogenotrophica*, which may instead employ OOR enzymes for oxalate degradation.

### 3.6. Expansion of Candidate Species Based on International Probiotic Lists

To enhance the generalizability and reference value of our study, we further cross-referenced the Qualified Presumption of Safety (QPS) list released by the European Food Safety Authority (Microbiological agents as notified to EFSA, March 2025 update, https://zenodo.org/records/15827510 accessed on 19 October 2025) and the probiotic lists published by the non-profit organization *AEProbio*, which categorize probiotics by health applications in food, adult health, and child health. From these sources, we identified seven additional species not previously included in our dataset: *Ligilactobacillus animalis* (*Lig. animalis*), *Lactobacillus amylovorus* (*L. amylovorus*), *Lentilactobacillus hilgardii*, *Lentilactobacillus buchneri*, *Lactobacillus farciminis*, *Lactobacillus brevis*, and *Lactiplantibacillus argentoratensis*. These species were subsequently analyzed following the same pipeline as described above.

**Figure 6:**
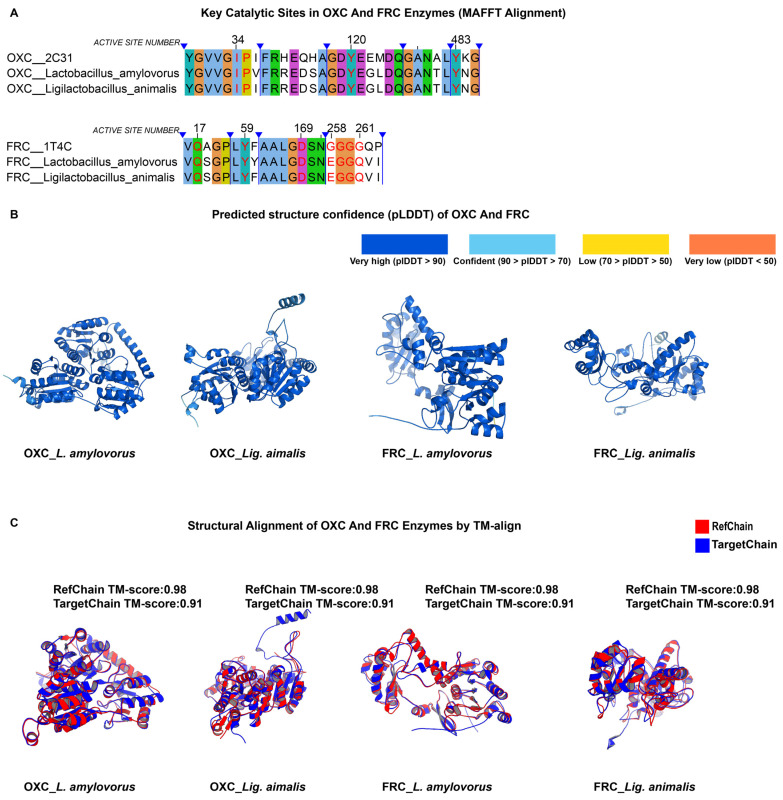
Comparative analysis of OXC and FRC enzymes in additional probiotic species identified from international probiotic lists (*EFSA QPS* and *AEProbio*). (**A**) In this figure legend, the meanings of the blue markings and background colors are the same as previously described. MSA (MAFFT) showing conservation of key catalytic residues in *L*. *amylovorus* and *Lig. animalis* relative to reference enzymes. (**B**) AlphaFold-predicted structures of OXC and FRC, with pLDDT indicating high model reliability (dark blue, pLDDT > 90). (**C**) Structural superposition of predicted and reference enzymes using TM-align. TM-scores > 0.9 indicate strong structural congruence.

As shown in Supplementary Table S6, the OXC and FRC proteins of *L. amylovorus* and *Lig. animalis* exhibited high sequence homology with the reference enzymes, both exceeding 44% identity, and greater than 70% identity when compared with *L. gasseri*. In Figure 6, MSA demonstrated strong conservation of catalytic residues in both species, with no mutations observed at key active sites. The AlphaFold prediction confidence (pLDDT) maps were predominantly blue, indicating high model reliability. Furthermore, TM-align results yielded TM-scores above 0.9 and RMSD values below 2 Å for both OXC and FRC proteins, suggesting excellent structural congruence with the reference enzymes.

Collectively, these findings are consistent with our prior results and indicate that *L. amylovorus* and *Lig. animalis* possess conserved enzymatic architectures and catalytic motifs characteristic of oxalate-degrading enzymes. These two species therefore represent promising candidates with potential oxalate-degrading activity.

## 4. Discussion

In this study, we systematically integrated genomic data from multiple probiotic species to investigate the distribution and conservation of oxalate-degrading genes. By selecting high-quality representative genomes and applying a series of bioinformatics analyses, we revealed species-level patterns of functional gene distribution and identified probiotic taxa with potential oxalate-degrading activity. Unlike most previous studies, which focused on individual strains, our work expands the analytical scope to the species level, thereby providing a broader perspective on the metabolic potential of probiotic populations in oxalate degradation. This approach not only supplements current knowledge of probiotic functionality but also offers practical guidance for selecting candidates for functional probiotic development.

Our findings align with previous reports on *L. gasseri*, which has been shown to degrade oxalate [47]. *B. animalis* was also predicted to have oxalate-degrading activity, consistent with studies demonstrating that *B. animalis* subsp. *lactis* DSM10140 reduces urinary oxalate excretion in a mouse model of primary hyperoxaluria [48]. Similarly, *Lactobacillus acidophilus* has repeatedly been reported to degrade oxalate, with one study showing up to a 98.86% ± 0.87 reduction in oxalate content [49]. In contrast, evidence for *Limosilactobacillus reuteri* remains limited, though comparative analyses suggest that certain isolates possess oxalate-degrading capacity [50]. For *L. kefiranofaciens*, data on oxalate metabolism are extremely sparse. Although our genomic analysis revealed the presence of both *OXC* and *FRC*, the truncated *FRC* gene raises the possibility of alternative or compensatory mechanisms that may restore enzymatic function. Likewise, *L. johnsonii* has not been directly linked to oxalate metabolism; our results suggest it may represent a previously unrecognized oxalate degrader, warranting further experimental validation.

Next-generation probiotics have primarily been studied for their roles in host metabolism and immunity [51,52], with little attention given to oxalate metabolism. In our analysis, *B. pseudococcoides* and *B. hydrogenotrophica* lacked *OXC* and *FRC* genes, and while *OOR* clusters were identified, structural and active-site analyses did not yield strong evidence of robust enzymatic activity. Thus, their oxalate-degrading potential remains speculative and requires direct experimental confirmation.

Taken together, our study expands the repertoire of potential oxalate-degrading probiotics. Moreover, incorporating species from international probiotic lists (*EFSA QPS* and *AEProbio*) yielded consistent findings, further supporting the robustness of our analytical framework. Among traditional species, we identified three underexplored candidates, including *L. johnsonii*, *L. kefiranofaciens* and *L. reuteri*, that likely rely on the OXC–FRC pathway. Notably, *L. kefiranofaciens* harbors two FRC-like sequences with complementary structural similarity to the reference enzyme, suggesting a potential but unverified oxalate-degrading capacity. Among next-generation probiotics, *B. pseudococcoides* and *B. hydrogenotrophica* emerged as putative oxalate degraders that may employ an alternative OOR pathway, although this prediction remains to be experimentally validated.

Beyond genomic predictions, the potential biological implications of oxalate-degrading probiotics warrant consideration. By degrading oxalate in the gut, these species may help maintain host oxalate balance, reduce intestinal oxalate absorption, and contribute to metabolic homeostasis. To confirm the predicted activity, future in vitro and in vivo studies could assess oxalate depletion or enzyme activity in culture and evaluate probiotic administration in animal models of hyperoxaluria. Such validation would provide direct evidence supporting the bioinformatic and structural findings of this study.

This work has several limitations. First, by focusing on high-quality representative genomes, we did not capture intraspecies variation, which may affect the distribution and function of oxalate-degrading genes. Second, as an in silico study, our findings remain predictive and require validation through in vitro and in vivo experiments. Future research should therefore explore strain-level diversity, evaluate transcriptional activity of candidate genes using transcriptomics, and confirm enzymatic activity through functional assays.

In summary, this study provides the first species-level overview of oxalate-degrading probiotics based on integrated genome and protein structure analyses. The workflow established here is scalable and reproducible, offering a useful framework for identifying probiotic candidates for functional evaluation and future therapeutic application.

## Data Availability

All genome data analyzed in this study are publicly available at National Center for Biotechnology Information (NCBI) database https://www.ncbi.nlm.nih.gov/ (accessed on 25 July 2025). Additional data supporting the findings of this study, including analysis scripts and processed datasets, are available from the corresponding author upon reasonable request.

## Supplementary Materials

The following supporting information can be downloaded at: https://www.mdpi.com/article/10.3390/microorganisms13112553/s1, Table S1: List of probiotic species analyzed in this study; Table S2: NCBI accession numbers of all genomes used in this study; Table S3: Reference protein sequences used for BLAST analysis; Table S4: BLAST Alignment-Based Homology Quantification Table with Reference Chain; Table S5: BLAST Alignment-Based Homology Quantification Table with *L. gasseri* as Internal Control; Table S6: Sequence homology data of additional probiotic species analyzed in the extended dataset.
