# Supplementary material for: Bioinformatic Analysis of Oxalate-Degrading Enzymes in Probiotics: A Systematic Genome-Scale and Structural Survey
Source: Microorganisms. 2025 Nov 8;13(11):2553. doi: 10.3390/microorganisms13112553 (PMC12654022; doi:10.3390/microorganisms13112553)
Supplement: Supplementary file 1 [file microorganisms-13-02553-s001.zip › Supplementary Table S1.pdf]

Table S1. List of probiotic species analyzed in this study.

Tradition Probiotics
Bifidobacterium adolescentis
Lactobacillus gasseri
Leuconostoc mesenteroides
Pediococcus pentosaceus
Bifidobacterium animalis
Lactobacillus delbrueckii
Propionibacterium freudenreichii
Lacticaseibacillus paracasei
Bifidobacterium longum
Bifidobacterium animalis
Lactococcus cremoris
Staphylococcus xylosus
Lacticaseibacillus casei
Heyndrickxia coagulans
Bifidobacterium breve
Kluyveromyces marxianus
Acidipropionibacterium acidipropionici
Lactobacillus delbrueckii
Lactococcus lactis
Lactococcus lactis
Limosilactobacillus reuteri
Latilactobacillus curvatus
Lacticaseibacillus rhamnosus
Lactobacillus kefiranofaciens
Latilactobacillus sakei
Lactobacillus crispatus
Lactiplantibacillus plantarum
Streptococcus thermophilus
Pediococcus acidilactici
Lactobacillus johnsonii
Staphylococcus carnosus
Mammaliicoccus vitulinus
Bifidobacterium bifidum
Limosilactobacillus fermentum
Lactobacillus acidophilus
Ligilactobacillus salivarius
Lactobacillus helveticus
Bifidobacterium longum

Next-generation Probiotics

Akkermansia muciniphila
Blautia acetigignens
Blautia ammoniilytica
Blautia argi
Blautia caecimuris
Blautia celeris
Blautia faecis
Blautia hansenii
Blautia hydrogenotrophica
Blautia intestinalis
Blautia liquoris
Blautia luti
Blautia marasmi
Blautia massiliensis
Blautia obeum
Blautia parvula
Blautia producta
Blautia pseudococcoides
Blautia sp.
Blautia wexlerae
Lachnospira eligens
Lachnospira multipara
Lachnospira pectinoschiza
Candidatus Borkfalkia
Candidatus Caballimonas
Candidatus Caccalectryoclostridium
Candidatus Onthoplasma
Candidatus Ornithoclostridium
Candidatus Spyradocola
Christensenella hongkongensis
Christensenella intestinihominis
Christensenella massiliensis
Christensenella minuta
Christensenella tenuis
Christensenella timonensis
Gehongia tenuis
Guopingia tenuis
Luoshenia tenuis
Bacteroides fragilis
Bacteroides xylanisolvens
Clostridium butyricum
Faecalibacterium prausnitzii
Parabacteroides distasonis
Parabacteroides merdae

Roseburia amylophila
Roseburia difficilis
Roseburia faecis
Roseburia hominis
Roseburia intestinalis
Roseburia inulinivorans
Roseburia lenta
Roseburia porci
Roseburia rectibacter
Roseburia sp.
Roseburia yibonii
Roseburia zhanii
uncultured Roseburia
Anaerobutyricum hallii
Bacteroides thetaiotaomicron
Parabacteroides goldsteinii
Prevotella corporis
