# Supplementary material for: Bioinformatic Analysis of Oxalate-Degrading Enzymes in Probiotics: A Systematic Genome-Scale and Structural Survey
Source: Microorganisms. 2025 Nov 8;13(11):2553. doi: 10.3390/microorganisms13112553 (PMC12654022; doi:10.3390/microorganisms13112553)
Supplement: Supplementary file 1 [file microorganisms-13-02553-s001.zip › Supplementary Table S2.pdf]

Table S2. NCBI accession numbers of all genomes used in this study.

NCBI Accession Number	Species Name
GCF_000010425.1	*Bifidobacterium adolescentis*
GCF_000014425.1	*Lactobacillus gasseri*
GCF_000014445.1	*Leuconostoc mesenteroides*
GCF_000014505.1	*Pediococcus pentosaceus*
GCF_000022965.1	*Bifidobacterium animalis*
GCF_000056065.1	*Lactobacillus delbrueckii*
GCF_000091725.1	*Propionibacterium freudenreichii*
GCF_000155515.2	*Lacticaseibacillus paracasei*
GCF_000196555.1	*Bifidobacterium longum*
GCF_000260715.1	*Bifidobacterium animalis*
GCF_000468955.1	*Lactococcus cremoris*
GCF_000709415.1	*Staphylococcus xylosus*
GCF_000829055.1	*Lacticaseibacillus casei*
GCF_000832905.1	*Heyndrickxia coagulans*
GCF_001025175.1	*Bifidobacterium breve*
GCF_001417885.1	*Kluyveromyces marxianus*
GCF_001441165.1	*Acidipropionibacterium acidipropionici*
GCF_002278095.1	*Lactobacillus delbrueckii*
GCF_003176835.1	*Lactococcus lactis*
GCF_003627395.2	*Lactococcus lactis*
GCF_003703885.1	*Limosilactobacillus reuteri*
GCF_004101845.1	*Latilactobacillus curvatus*
GCF_006151905.1	*Lacticaseibacillus rhamnosus*
GCF_009184665.1	*Lactobacillus kefiranofaciens*
GCF_009676365.1	*Latilactobacillus sakei*
GCF_009769205.1	*Lactobacillus crispatus*
GCF_009913655.1	*Lactiplantibacillus plantarum*
GCF_010120595.1	*Streptococcus thermophilus*
GCF_013127755.1	*Pediococcus acidilactici*
GCF_014058685.1	*Lactobacillus johnsonii*
GCF_016028275.1	*Staphylococcus carnosus*
GCF_016725245.1	*Mammaliicoccus vitulinus*
GCF_017894325.1	*Bifidobacterium bifidum*
GCF_027668665.1	*Limosilactobacillus fermentum*
GCF_034298135.1	*Lactobacillus acidophilus*
GCF_035231985.1	*Ligilactobacillus salivarius*
GCF_046109915.1	*Lactobacillus helveticus*
GCF_900637215.1	*Bifidobacterium longum*
GCF_018389265.1	*Lactobacillus gasseri*
GCF_030418275.1	*Limosilactobacillus reuteri*
GCF_036903235.1	*Limosilactobacillus reuteri*
GCF_046763525.1	*Limosilactobacillus reuteri*

GCF_001889975.1	*Limosilactobacillus reuteri*
GCF_004683835.1	*Limosilactobacillus fermentum*
GCF_012070605.1	*Limosilactobacillus fermentum*
GCF_024385625.1	*Limosilactobacillus fermentum*
GCF_025191645.1	*Limosilactobacillus fermentum*
GCF_027668665.1	*Limosilactobacillus fermentum*
GCF_027681185.1	*Limosilactobacillus fermentum*
GCF_037006085.1	*Limosilactobacillus fermentum*
GCF_049180395.1	*Limosilactobacillus fermentum*
GCF_049180455.1	*Limosilactobacillus fermentum*
GCF_902363965.1	*Limosilactobacillus fermentum*
GCF_001020245.1	*Bifidobacterium bifidum*
GCF_001020255.1	*Bifidobacterium bifidum*
GCF_001020355.1	*Bifidobacterium bifidum*
GCF_001020375.1	*Bifidobacterium bifidum*
GCF_009077915.1	*Bifidobacterium bifidum*
GCF_020710245.1	*Bifidobacterium bifidum*
GCF_020861855.1	*Bifidobacterium bifidum*
GCF_022134905.2	*Bifidobacterium bifidum*
GCF_026162505.1	*Bifidobacterium bifidum*
GCF_051063355.1	*Bifidobacterium bifidum*
GCF_051063555.1	*Bifidobacterium bifidum*
GCF_009808645.1	*Pediococcus pentosaceus*
GCF_015613015.1	*Pediococcus pentosaceus*
GCF_015613165.1	*Pediococcus pentosaceus*
GCF_015613205.1	*Pediococcus pentosaceus*
GCF_015613235.1	*Pediococcus pentosaceus*
GCF_015613245.1	*Pediococcus pentosaceus*
GCF_016652195.1	*Pediococcus pentosaceus*
GCF_029439595.1	*Pediococcus pentosaceus*
GCF_034321905.1	*Pediococcus pentosaceus*
GCF_044901865.1	*Pediococcus pentosaceus*
GCF_006538245.1	*Bifidobacterium animalis*
GCF_006538265.1	*Bifidobacterium animalis*
GCF_009075825.1	*Bifidobacterium animalis*
GCF_009077835.1	*Bifidobacterium animalis*
GCF_018408985.1	*Bifidobacterium animalis*
GCF_018409015.1	*Bifidobacterium animalis*
GCF_027659385.1	*Bifidobacterium animalis*
GCF_027659815.1	*Bifidobacterium animalis*
GCF_027659965.1	*Bifidobacterium animalis*
GCF_027662985.1	*Bifidobacterium animalis*
GCF_027663025.1	*Bifidobacterium animalis*
GCF_027690475.1	*Bifidobacterium animalis*

GCF_039503425.1	*Bifidobacterium animalis*
GCF_005049195.1	*Latilactobacillus curvatus*
GCF_024272865.1	*Latilactobacillus curvatus*
GCF_024272885.1	*Latilactobacillus curvatus*
GCF_024272925.1	*Latilactobacillus curvatus*
GCF_024272965.1	*Latilactobacillus curvatus*
GCF_024273015.1	*Latilactobacillus curvatus*
GCF_025193945.1	*Latilactobacillus curvatus*
GCF_025194065.1	*Latilactobacillus curvatus*
GCF_029581685.1	*Latilactobacillus curvatus*
GCF_029581735.1	*Latilactobacillus curvatus*
GCF_029581755.1	*Latilactobacillus curvatus*
GCF_965118885.1	*Latilactobacillus curvatus*
GCF_000771305.1	*Bifidobacterium breve*
GCF_016648955.1	*Bifidobacterium breve*
GCF_027212565.1	*Bifidobacterium breve*
GCF_027212815.1	*Bifidobacterium breve*
GCF_027214675.1	*Bifidobacterium breve*
GCF_027214825.1	*Bifidobacterium breve*
GCF_027215465.1	*Bifidobacterium breve*
GCF_027215475.1	*Bifidobacterium breve*
GCF_050255715.1	*Bifidobacterium breve*
GCF_050255735.1	*Bifidobacterium breve*
GCF_051063395.1	*Bifidobacterium breve*
GCF_051063615.1	*Bifidobacterium breve*
GCF_902651995.1	*Bifidobacterium breve*
GCF_000310065.1	*Acidipropionibacterium acidipropionici*
GCF_000427845.1	*Acidipropionibacterium acidipropionici*
GCF_001602115.1	*Acidipropionibacterium acidipropionici*
GCF_001855335.1	*Acidipropionibacterium acidipropionici*
GCF_001975205.1	*Acidipropionibacterium acidipropionici*
GCF_003956085.1	*Acidipropionibacterium acidipropionici*
GCF_005890155.1	*Acidipropionibacterium acidipropionici*
GCF_036452765.1	*Acidipropionibacterium acidipropionici*
GCF_036452805.1	*Acidipropionibacterium acidipropionici*
GCF_001051015.2	*Bifidobacterium longum*
GCF_001281305.1	*Bifidobacterium longum*
GCF_001293145.1	*Bifidobacterium longum*
GCF_001446255.1	*Bifidobacterium longum*
GCF_011764605.1	*Bifidobacterium longum*
GCF_013204895.1	*Bifidobacterium longum*
GCF_013249165.1	*Bifidobacterium longum*
GCF_013393765.1	*Bifidobacterium longum*
GCF_014334075.1	*Bifidobacterium longum*

GCF_014334375.1	*Bifidobacterium longum*
GCF_000169195.2	*Heyndrickxia coagulans*
GCF_001578455.1	*Heyndrickxia coagulans*
GCF_001870065.1	*Heyndrickxia coagulans*
GCF_015547595.1	*Heyndrickxia coagulans*
GCF_016724825.1	*Heyndrickxia coagulans*
GCF_017353475.1	*Heyndrickxia coagulans*
GCF_018603675.1	*Heyndrickxia coagulans*
GCF_018603695.1	*Heyndrickxia coagulans*
GCF_019443305.1	*Heyndrickxia coagulans*
GCF_021764685.1	*Heyndrickxia coagulans*
GCF_001591705.1	*Lactococcus cremoris*
GCF_032586255.1	*Lactococcus cremoris*
GCF_036670465.1	*Lactococcus cremoris*
GCF_036670495.1	*Lactococcus cremoris*
GCF_036670575.1	*Lactococcus cremoris*
GCF_036670585.1	*Lactococcus cremoris*
GCF_036670615.1	*Lactococcus cremoris*
GCF_036670625.1	*Lactococcus cremoris*
GCF_036670665.1	*Lactococcus cremoris*
GCF_036670705.1	*Lactococcus cremoris*
GCF_000154085.1	*Bifidobacterium adolescentis*
GCF_015547885.1	*Bifidobacterium adolescentis*
GCF_015548665.1	*Bifidobacterium adolescentis*
GCF_015548755.1	*Bifidobacterium adolescentis*
GCF_015548985.1	*Bifidobacterium adolescentis*
GCF_015549865.1	*Bifidobacterium adolescentis*
GCF_015552825.1	*Bifidobacterium adolescentis*
GCF_015553845.1	*Bifidobacterium adolescentis*
GCF_015553925.1	*Bifidobacterium adolescentis*
GCA_000981035.1	*Christensenella hongkongensis*
GCA_001507385.1	*Christensenella hongkongensis*
GCA_001571425.1	*Christensenella minuta*
GCA_001652705.1	*Christensenella minuta*
GCA_001678845.1	*Christensenella intestinihominis*
GCA_001678855.1	*Christensenella minuta*
GCA_003628755.1	*Christensenella minuta*
GCA_004134775.1	*Candidatus Borkfalkia*
GCA_004342745.1	*Christensenella hongkongensis*
GCA_014287795.1	*Christensenella tenuis*
GCA_014384745.1	*Luoshenia tenuis*
GCA_014384795.1	*Gehongia tenuis*
GCA_014384805.1	*Guopingia tenuis*
GCA_018065575.1	*Candidatus Caballimonas*

GCA_018711265.1	*Candidatus Spyradocola*
GCA_018713665.1	*Candidatus Ornithoclostridium*
GCA_018714045.1	*Candidatus Ornithoclostridium*
GCA_018714485.1	*Candidatus Caccalectryoclostridium*
GCA_018715225.1	*Candidatus Onthoplasma*
GCA_018716085.1	*Candidatus Ornithoclostridium*
GCA_019114245.1	*Candidatus Borkfalkia*
GCA_019114625.1	*Candidatus Borkfalkia*
GCA_019120295.1	*Candidatus Onthoplasma*
GCA_019420825.1	*Christensenella massiliensis*
GCA_024706965.1	*Christensenella minuta*
GCA_027680515.1	*Christensenella intestinihominis*
GCA_030825545.1	*Candidatus Borkfalkia*
GCA_034090205.1	*Christensenella hongkongensis*
GCA_034099915.1	*Christensenella minuta*
GCA_037297545.1	*Candidatus Borkfalkia*
GCA_039778295.1	*Christensenella minuta*
GCA_039830115.1	*Christensenella minuta*
GCA_040409835.1	*Christensenella massiliensis*
GCA_048176945.1	*Candidatus Borkfalkia*
GCA_048341395.1	*Candidatus Spyradocola*
GCA_048357735.1	*Candidatus Spyradocola*
GCA_048419665.1	*Candidatus Borkfalkia*
GCA_050945035.1	*Luoshenia tenuis*
GCA_900087015.1	*Christensenella timonensis*
GCA_902376065.1	*Christensenella timonensis*
GCA_902388075.1	*Christensenella minuta*
GCA_937923295.1	*Candidatus Borkfalkia*
GCA_944328435.1	*Candidatus Borkfalkia*
GCA_944340515.1	*Candidatus Borkfalkia*
GCA_944340995.1	*Candidatus Borkfalkia*
GCA_944380345.1	*Candidatus Onthoplasma*
GCA_944380385.1	*Candidatus Spyradocola*
GCA_944381285.1	*Candidatus Caccalectryoclostridium*
GCA_944384345.1	*Candidatus Ornithoclostridium*
GCA_944384355.1	*Candidatus Ornithoclostridium*
GCA_944384365.1	*Candidatus Ornithoclostridium*
GCA_944384435.1	*Candidatus Onthoplasma*
GCA_947853995.1	*Candidatus Ornithoclostridium*
GCA_947856195.1	*Candidatus Caccalectryoclostridium*
GCA_958346725.1	*Christensenella minuta*
GCA_958441125.1	*Candidatus Borkfalkia*
GCA_959020245.1	*Christensenella hongkongensis*
GCA_963534795.1	*Christensenella hongkongensis*

GCA_963927015.1	*Candidatus Caccalectryoclostridium*
GCA_963927895.1	*Candidatus Borkfalkia*
GCA_963931195.1	*Candidatus Borkfalkia*
GCF_000020225.1	*Akkermansia muciniphila*
GCF_000146185.1	*Lachnospira eligens*
GCF_000153905.1	*Blautia obeum*
GCF_000156675.1	*Blautia hansenii*
GCF_000157975.1	*Blautia hydrogenotrophica*
GCF_000210015.1	*Blautia obeum*
GCF_000373885.1	*Blautia producta*
GCF_000424085.1	*Blautia wexlerae*
GCF_000424105.1	*Lachnospira multipara*
GCF_000431095.1	*Blautia hydrogenotrophica*
GCF_000439125.1	*Blautia producta*
GCF_000466565.1	*Blautia sp.*
GCF_000484655.1	*Blautia wexlerae*
GCF_000621945.1	*Lachnospira multipara*
GCF_000702205.1	*Lachnospira multipara*
GCF_001404435.1	*Lachnospira eligens*
GCF_001404455.1	*Blautia obeum*
GCF_001404535.1	*Blautia obeum*
GCF_001404735.1	*Blautia wexlerae*
GCF_001404755.1	*Blautia wexlerae*
GCF_001404775.1	*Blautia obeum*
GCF_001404935.1	*Blautia hydrogenotrophica*
GCF_001405215.1	*Blautia obeum*
GCF_001405395.1	*Lachnospira eligens*
GCF_001405455.1	*Blautia obeum*
GCF_001487165.1	*Blautia massiliensis*
GCF_001689125.2	*Blautia pseudococcoides*
GCF_002221555.2	*Blautia pseudococcoides*
GCF_002222595.2	*Blautia hansenii*
GCF_003287895.1	*Blautia argi*
GCF_003435675.1	*Blautia sp.*
GCF_003460195.1	*Lachnospira eligens*
GCF_003460565.1	*Blautia sp.*
GCF_003460955.1	*Blautia sp.*
GCF_003461245.1	*Blautia sp.*
GCF_003464175.1	*Lachnospira eligens*
GCF_003464645.1	*Blautia obeum*
GCF_003465235.1	*Blautia obeum*
GCF_003465585.1	*Lachnospira eligens*
GCF_003466965.1	*Blautia obeum*
GCF_003466985.1	*Lachnospira eligens*

GCF_003467325.1	*Blautia obeum*
GCF_003467455.1	*Blautia obeum*
GCF_003467675.1	*Blautia obeum*
GCF_003467705.1	*Lachnospira eligens*
GCF_003470215.1	*Lachnospira eligens*
GCF_003470555.1	*Blautia obeum*
GCF_003471125.1	*Blautia obeum*
GCF_003473445.1	*Lachnospira eligens*
GCF_003474255.1	*Blautia obeum*
GCF_003474705.1	*Blautia obeum*
GCF_003474735.1	*Lachnospira eligens*
GCF_003474935.1	*Lachnospira eligens*
GCF_003475275.1	*Blautia obeum*
GCF_003488505.1	*Blautia sp.*
GCF_004104435.1	*Akkermansia muciniphila*
GCF_004210255.1	*Blautia producta*
GCF_004340925.1	*Blautia producta*
GCF_005845175.1	*Blautia obeum*
GCF_005848555.1	*Blautia sp.*
GCF_008000975.1	*Akkermansia muciniphila*
GCF_009680455.1	*Lachnospira eligens*
GCF_009731575.1	*Akkermansia muciniphila*
GCF_009876855.1	*Blautia massiliensis*
GCF_009876865.1	*Blautia wexlerae*
GCF_009876875.1	*Blautia massiliensis*
GCF_009881235.1	*Blautia wexlerae*
GCF_009881255.1	*Blautia wexlerae*
GCF_009883055.1	*Blautia sp.*
GCF_009883065.1	*Blautia obeum*
GCF_009883115.1	*Blautia wexlerae*
GCF_010223095.1	*Akkermansia muciniphila*
GCF_010509235.1	*Akkermansia muciniphila*
GCF_010669205.1	*Blautia producta*
GCF_013112015.1	*Blautia pseudococcoides*
GCF_014131715.1	*Blautia producta*
GCF_014170075.1	*Akkermansia muciniphila*
GCF_015159595.1	*Blautia liquoris*
GCF_015549355.1	*Blautia wexlerae*
GCF_015549855.1	*Blautia massiliensis*
GCF_015553285.1	*Lachnospira eligens*
GCF_015554555.1	*Blautia wexlerae*
GCF_015556425.1	*Blautia wexlerae*
GCF_015559345.1	*Lachnospira eligens*
GCF_015559385.1	*Blautia massiliensis*

GCF_015560815.1	*Blautia massiliensis*
GCF_015561285.1	*Blautia wexlerae*
GCF_015667435.1	*Blautia obeum*
GCF_015667495.1	*Blautia wexlerae*
GCF_015668015.1	*Blautia obeum*
GCF_015668165.1	*Blautia sp.*
GCF_015668375.1	*Blautia wexlerae*
GCF_015669135.1	*Blautia luti*
GCF_015669755.1	*Blautia wexlerae*
GCF_015670615.1	*Blautia wexlerae*
GCF_016696745.1	*Blautia pseudococcoides*
GCF_017504145.1	*Akkermansia muciniphila*
GCF_018785545.1	*Blautia wexlerae*
GCF_018785565.1	*Blautia sp.*
GCF_018785605.1	*Blautia sp.*
GCF_018785625.1	*Blautia faecis*
GCF_018785655.1	*Blautia sp.*
GCF_018847215.1	*Akkermansia muciniphila*
GCF_018847255.1	*Akkermansia muciniphila*
GCF_018847315.1	*Akkermansia muciniphila*
GCF_018847335.1	*Akkermansia muciniphila*
GCF_018847355.1	*Akkermansia muciniphila*
GCF_018847395.1	*Akkermansia muciniphila*
GCF_018847535.1	*Akkermansia muciniphila*
GCF_019413035.1	*Blautia sp.*
GCF_019413225.1	*Blautia sp.*
GCF_019424595.1	*Blautia sp.*
GCF_020215665.1	*Blautia parvula*
GCF_020735365.1	*Blautia massiliensis*
GCF_020735745.1	*Lachnospira eligens*
GCF_023656705.1	*Blautia sp.*
GCF_025147655.1	*Blautia hansenii*
GCF_025147765.1	*Blautia obeum*
GCF_025147905.1	*Blautia sp.*
GCF_025148125.1	*Blautia wexlerae*
GCF_025289255.1	*Blautia hydrogenotrophica*
GCF_025566865.1	*Blautia acetigignens*
GCF_025566965.1	*Blautia ammoniilytica*
GCF_027660665.1	*Blautia wexlerae*
GCF_027661825.1	*Blautia obeum*
GCF_027661925.1	*Blautia massiliensis*
GCF_027662005.1	*Blautia wexlerae*
GCF_027662215.1	*Blautia wexlerae*
GCF_027662325.1	*Blautia faecis*

GCF_027663385.1	*Blautia faecis*
GCF_027663885.1	*Blautia intestinalis*
GCF_027663965.1	*Blautia massiliensis*
GCF_027664185.1	*Blautia wexlerae*
GCF_027664225.1	*Blautia massiliensis*
GCF_027664245.1	*Blautia obeum*
GCF_027665425.1	*Blautia obeum*
GCF_027666705.1	*Blautia intestinalis*
GCF_027668025.1	*Blautia massiliensis*
GCF_027671005.1	*Blautia obeum*
GCF_027671065.1	*Blautia hansenii*
GCF_027671365.1	*Blautia massiliensis*
GCF_027697505.1	*Blautia massiliensis*
GCF_028210795.1	*Blautia wexlerae*
GCF_028314285.1	*Blautia wexlerae*
GCF_028314305.1	*Blautia wexlerae*
GCF_028743255.1	*Akkermansia muciniphila*
GCF_032142795.1	*Blautia producta*
GCF_032142815.1	*Blautia faecis*
GCF_033096465.1	*Blautia luti*
GCF_034355335.1	*Blautia producta*
GCF_034356035.1	*Blautia hydrogenotrophica*
GCF_039031535.1	*Blautia producta*
GCF_039067065.1	*Blautia obeum*
GCF_039503395.1	*Blautia celeris*
GCF_039503415.1	*Blautia wexlerae*
GCF_039753515.1	*Blautia wexlerae*
GCF_039753535.1	*Blautia obeum*
GCF_039837395.1	*Blautia wexlerae*
GCF_039837405.1	*Blautia wexlerae*
GCF_039837435.1	*Blautia wexlerae*
GCF_040910625.1	*Blautia obeum*
GCF_040910705.1	*Blautia sp.*
GCF_040929965.1	*Blautia sp.*
GCF_041222945.1	*Blautia producta*
GCF_041223265.1	*Blautia producta*
GCF_041223525.1	*Blautia marasmi*
GCF_041225785.1	*Blautia pseudococcoides*
GCF_041941475.1	*Blautia producta*
GCF_042847785.1	*Blautia wexlerae*
GCF_042852025.1	*Blautia parvula*
GCF_042853075.1	*Blautia producta*
GCF_042854155.1	*Blautia producta*
GCF_046603735.1	*Blautia producta*

GCF_046603745.1	*Blautia hansenii*
GCF_046603795.1	*Blautia obeum*
GCF_046603905.1	*Blautia producta*
GCF_046886425.1	*Blautia massiliensis*
GCF_046944355.1	*Blautia massiliensis*
GCF_046952935.1	*Blautia massiliensis*
GCF_003482385.1	*Blautia sp.*
GCF_046957695.1	*Blautia sp.*
GCF_046962975.1	*Blautia massiliensis*
GCF_046972005.1	*Blautia sp.*
GCF_046972825.1	*Blautia sp.*
GCF_046981945.1	*Blautia massiliensis*
GCF_046982505.1	*Blautia caecimuris*
GCF_046986205.1	*Blautia massiliensis*
GCF_047031475.1	*Blautia massiliensis*
GCF_047031575.1	*Blautia hydrogenotrophica*
GCF_047234555.1	*Blautia sp.*
GCF_047238505.1	*Blautia massiliensis*
GCF_047241565.1	*Blautia massiliensis*
GCF_047245205.1	*Blautia massiliensis*
GCF_047247325.1	*Blautia massiliensis*
GCF_900078295.2	*Blautia sp.*
GCF_900101315.1	*Blautia sp.*
GCF_900103815.1	*Lachnospira pectinoschiza*
GCF_900108125.1	*Lachnospira multipara*
GCF_900120195.1	*Blautia sp.*
GCF_900120295.1	*Blautia sp.*
GCF_902362465.1	*Blautia hydrogenotrophica*
GCF_902362785.1	*Lachnospira eligens*
GCF_902364415.1	*Blautia obeum*
GCF_902364805.1	*Blautia obeum*
GCF_902377405.1	*Blautia producta*
GCF_934882065.1	*Blautia massiliensis*
GCF_964237855.1	*Blautia massiliensis*
GCF_964238075.1	*Blautia obeum*
GCF_964238905.1	*Blautia obeum*
GCF_964240315.1	*Blautia sp.*
GCF_964240825.1	*Blautia obeum*
GCF_964241055.1	*Blautia producta*
GCF_964287955.1	*Blautia luti*
GCF_000012845.1	*Parabacteroides distasonis*
GCF_000025985.1	*Bacteroides fragilis*
GCF_000154105.1	*Parabacteroides merdae*
GCF_000154385.1	*Faecalibacterium prausnitzii*

GCF_000210075.1	*Bacteroides xylanisolvens*
GCF_000710365.2	*Bacteroides fragilis*
GCF_000710375.2	*Bacteroides fragilis*
GCF_001404575.1	*Parabacteroides merdae*
GCF_001456065.2	*Clostridium butyricum*
GCF_001465175.1	*Clostridium butyricum*
GCF_001646605.1	*Clostridium butyricum*
GCF_001886875.1	*Clostridium butyricum*
GCF_002586945.1	*Faecalibacterium prausnitzii*
GCF_002849695.1	*Bacteroides fragilis*
GCF_003312465.1	*Faecalibacterium prausnitzii*
GCF_003470675.1	*Parabacteroides merdae*
GCF_004166975.1	*Parabacteroides merdae*
GCF_005145085.1	*Clostridium butyricum*
GCF_005706655.1	*Bacteroides fragilis*
GCF_006149185.1	*Parabacteroides distasonis*
GCF_006546965.1	*Bacteroides xylanisolvens*
GCF_006739545.1	*Parabacteroides distasonis*
GCF_006742065.1	*Clostridium butyricum*
GCF_007992895.1	*Clostridium butyricum*
GCF_008369705.1	*Bacteroides fragilis*
GCF_008710235.1	*Bacteroides xylanisolvens*
GCF_009650315.1	*Clostridium butyricum*
GCF_009650335.1	*Clostridium butyricum*
GCF_009719625.1	*Parabacteroides merdae*
GCF_012851305.1	*Parabacteroides distasonis*
GCF_013112415.1	*Clostridium butyricum*
GCF_013267555.1	*Bacteroides fragilis*
GCF_014131795.1	*Clostridium butyricum*
GCF_015554045.1	*Parabacteroides merdae*
GCF_015670365.1	*Parabacteroides merdae*
GCF_016864215.1	*Bacteroides xylanisolvens*
GCF_016864615.1	*Bacteroides fragilis*
GCF_018140655.1	*Clostridium butyricum*
GCF_018279805.1	*Bacteroides xylanisolvens*
GCF_018279895.1	*Parabacteroides distasonis*
GCF_018288975.1	*Parabacteroides distasonis*
GCF_018289035.1	*Bacteroides xylanisolvens*
GCF_018289135.1	*Bacteroides xylanisolvens*
GCF_018289315.1	*Parabacteroides merdae*
GCF_018289335.1	*Parabacteroides distasonis*
GCF_019967955.1	*Faecalibacterium prausnitzii*
GCF_019967975.1	*Faecalibacterium prausnitzii*
GCF_019968055.1	*Faecalibacterium prausnitzii*

GCF_020091365.1	*Parabacteroides merdae*
GCF_020091445.1	*Parabacteroides distasonis*
GCF_020097275.1	*Bacteroides fragilis*
GCF_020256765.1	*Bacteroides xylanisolvens*
GCF_020257025.1	*Bacteroides xylanisolvens*
GCF_020257245.1	*Bacteroides xylanisolvens*
GCF_020257345.1	*Bacteroides xylanisolvens*
GCF_020735605.1	*Parabacteroides merdae*
GCF_020735945.1	*Parabacteroides distasonis*
GCF_021359565.1	*Bacteroides fragilis*
GCF_022835275.1	*Parabacteroides merdae*
GCF_023702735.1	*Bacteroides fragilis*
GCF_024125345.1	*Parabacteroides merdae*
GCF_024399875.1	*Clostridium butyricum*
GCF_024463155.2	*Parabacteroides merdae*
GCF_024592595.1	*Parabacteroides merdae*
GCF_024758245.1	*Parabacteroides distasonis*
GCF_024758345.1	*Bacteroides xylanisolvens*
GCF_024759125.1	*Bacteroides xylanisolvens*
GCF_024759425.1	*Parabacteroides distasonis*
GCF_024759485.1	*Parabacteroides distasonis*
GCF_024759585.1	*Parabacteroides distasonis*
GCF_024789605.1	*Bacteroides xylanisolvens*
GCF_024794065.1	*Bacteroides xylanisolvens*
GCF_025151215.1	*Parabacteroides merdae*
GCF_025506235.1	*Bacteroides xylanisolvens*
GCF_026651935.1	*Clostridium butyricum*
GCF_027627495.1	*Clostridium butyricum*
GCF_028743395.1	*Faecalibacterium prausnitzii*
GCF_029369685.1	*Parabacteroides distasonis*
GCF_029369765.1	*Bacteroides xylanisolvens*
GCF_030389005.1	*Clostridium butyricum*
GCF_030758275.1	*Clostridium butyricum*
GCF_030875795.1	*Parabacteroides distasonis*
GCF_030944245.1	*Bacteroides fragilis*
GCF_036419355.1	*Parabacteroides distasonis*
GCF_039789235.1	*Parabacteroides distasonis*
GCF_040026365.1	*Faecalibacterium prausnitzii*
GCF_040059215.1	*Faecalibacterium prausnitzii*
GCF_040687795.1	*Bacteroides fragilis*
GCF_040687805.1	*Bacteroides fragilis*
GCF_041937605.1	*Clostridium butyricum*
GCF_042847385.1	*Parabacteroides merdae*
GCF_042850145.1	*Bacteroides xylanisolvens*

GCF_042850405.1	*Parabacteroides merdae*
GCF_042851245.1	*Bacteroides xylanisolvens*
GCF_047309225.1	*Parabacteroides merdae*
GCF_047946905.1	*Faecalibacterium prausnitzii*
GCF_048396385.1	*Bacteroides xylanisolvens*
GCF_049532955.1	*Faecalibacterium prausnitzii*
GCF_049991395.1	*Clostridium butyricum*
GCF_050366995.1	*Clostridium butyricum*
GCF_050502955.1	*Faecalibacterium prausnitzii*
GCF_051201525.1	*Faecalibacterium prausnitzii*
GCF_900445495.1	*Parabacteroides merdae*
GCF_902362625.1	*Parabacteroides merdae*
GCF_951792925.1	*Faecalibacterium prausnitzii*
GCF_000156535.1	*Roseburia intestinalis*
GCF_000174195.1	*Roseburia inulinivorans*
GCF_000209995.1	*Roseburia intestinalis*
GCF_000210655.1	*Roseburia intestinalis*
GCF_000225345.1	*Roseburia hominis*
GCF_000432035.1	*Roseburia inulinivorans*
GCF_000432675.1	*Roseburia sp.*
GCF_000435135.1	*Roseburia intestinalis*
GCF_000436955.1	*Roseburia sp.*
GCF_000437095.1	*Roseburia sp.*
GCF_000437755.1	*Roseburia sp.*
GCF_001405535.1	*Roseburia inulinivorans*
GCF_001405615.1	*Roseburia faecis*
GCF_001405875.1	*Roseburia hominis*
GCF_001406435.1	*Roseburia intestinalis*
GCF_001406675.1	*Roseburia inulinivorans*
GCF_001406815.1	*Roseburia faecis*
GCF_001406855.1	*Roseburia inulinivorans*
GCF_001940165.2	*Roseburia sp.*
GCF_001940225.2	*Roseburia sp.*
GCF_003435965.1	*Roseburia sp.*
GCF_003457795.1	*Roseburia inulinivorans*
GCF_003458535.1	*Roseburia inulinivorans*
GCF_003458765.1	*Roseburia hominis*
GCF_003458815.1	*Roseburia inulinivorans*
GCF_003462645.1	*Roseburia sp.*
GCF_003463035.1	*Roseburia sp.*
GCF_003467035.1	*Roseburia intestinalis*
GCF_003467265.1	*Roseburia inulinivorans*
GCF_003467725.1	*Roseburia intestinalis*
GCF_003470905.1	*Roseburia sp.*

GCF_003471045.1	*Roseburia intestinalis*
GCF_003473825.1	*Roseburia sp.*
GCF_003474885.1	*Roseburia intestinalis*
GCF_003475515.1	*Roseburia intestinalis*
GCF_003482195.1	*Roseburia sp.*
GCF_003612565.1	*Roseburia sp.*
GCF_005845255.1	*Roseburia hominis*
GCF_009695765.1	*Roseburia porci*
GCF_009718345.1	*Roseburia intestinalis*
GCF_009718405.1	*Roseburia faecis*
GCF_009718425.1	*Roseburia faecis*
GCF_009759525.1	*Roseburia intestinalis*
GCF_009917385.1	*Roseburia sp.*
GCF_013300445.1	*Roseburia intestinalis*
GCF_014287435.1	*Roseburia lenta*
GCF_014287515.2	*Roseburia rectibacter*
GCF_014287625.1	*Roseburia difficilis*
GCF_014287635.1	*Roseburia zhanii*
GCF_014297335.1	*Roseburia yibonii*
GCF_014864765.1	*Roseburia inulinivorans*
GCF_015549075.1	*Roseburia faecis*
GCF_015550045.1	*Roseburia faecis*
GCF_015554785.1	*Roseburia intestinalis*
GCF_015555785.1	*Roseburia intestinalis*
GCF_015556245.1	*Roseburia faecis*
GCF_015558055.1	*Roseburia faecis*
GCF_018365315.1	*Roseburia hominis*
GCF_018783985.1	*Roseburia hominis*
GCF_018784025.1	*Roseburia hominis*
GCF_018784035.1	*Roseburia inulinivorans*
GCF_018918225.1	*Roseburia sp.*
GCF_019418905.1	*Roseburia sp.*
GCF_020537425.1	*Roseburia faecis*
GCF_020557615.1	*Roseburia faecis*
GCF_020687525.1	*Roseburia amylophila*
GCF_020687575.1	*Roseburia sp.*
GCF_020731525.1	*Roseburia inulinivorans*
GCF_021531695.1	*Roseburia hominis*
GCF_023369655.1	*Roseburia hominis*
GCF_023497585.1	*Roseburia intestinalis*
GCF_025151715.1	*Roseburia intestinalis*
GCF_025567465.1	*Roseburia amylophila*
GCF_027668125.1	*Roseburia intestinalis*
GCF_039060385.1	*Roseburia faecis*

GCF_040092275.1	*Roseburia amylophila*
GCF_040094215.1	*Roseburia intestinalis*
GCF_040094765.1	*Roseburia amylophila*
GCF_040094825.1	*Roseburia amylophila*
GCF_040095095.1	*Roseburia inulinivorans*
GCF_040095355.1	*Roseburia faecis*
GCF_040095515.1	*Roseburia inulinivorans*
GCF_040226695.1	*Roseburia faecis*
GCF_040919265.1	*Roseburia hominis*
GCF_041325975.1	*Roseburia sp.*
GCF_041331025.1	*Roseburia hominis*
GCF_041333795.1	*Roseburia intestinalis*
GCF_045061065.1	*Roseburia faecis*
GCF_046965465.1	*Roseburia inulinivorans*
GCF_047009705.1	*Roseburia hominis*
GCF_048338705.1	*Roseburia hominis*
GCF_048426205.1	*Roseburia inulinivorans*
GCF_048524305.1	*Roseburia hominis*
GCF_049273875.1	*Roseburia faecis*
GCF_900537995.1	*Roseburia intestinalis*
GCF_902362885.1	*Roseburia intestinalis*
GCF_902364955.1	*Roseburia inulinivorans*
GCF_902387955.1	*Roseburia hominis*
GCF_902652455.1	*Roseburia intestinalis*
GCF_928721605.1	*Roseburia hominis*
GCF_934416025.1	*uncultured Roseburia*
GCF_934831275.1	*Roseburia sp.*
GCF_934882475.1	*Roseburia sp.*
GCF_937921265.1	*Roseburia inulinivorans*
GCF_938024035.1	*uncultured Roseburia*
GCF_958348555.1	*Roseburia hominis*
GCF_958352065.1	*Roseburia inulinivorans*
GCF_958352295.1	*Roseburia hominis*
GCF_958408675.1	*Roseburia inulinivorans*
GCF_958435485.1	*Roseburia hominis*
GCF_959598315.1	*Roseburia inulinivorans*
GCF_965142365.1	*Roseburia hominis*
GCF_965149475.1	*Roseburia inulinivorans*
GCF_000011065.1	*Bacteroides thetaiotaomicron*
GCF_000173975.1	*Anaerobutyricum hallii*
GCF_000430525.1	*Prevotella corporis*
GCF_000613365.1	*Prevotella corporis*
GCF_000969835.1	*Parabacteroides goldsteinii*
GCF_001314975.1	*Bacteroides thetaiotaomicron*

GCF_001405175.1	*Anaerobutyricum hallii*
GCF_001406155.1	*Anaerobutyricum hallii*
GCF_001546595.1	*Prevotella corporis*
GCF_003466185.1	*Anaerobutyricum hallii*
GCF_003466615.1	*Anaerobutyricum hallii*
GCF_003468475.1	*Anaerobutyricum hallii*
GCF_003473385.1	*Anaerobutyricum hallii*
GCF_003475545.1	*Anaerobutyricum hallii*
GCF_003670235.1	*Parabacteroides goldsteinii*
GCF_014131755.1	*Bacteroides thetaiotaomicron*
GCF_015234935.1	*Parabacteroides goldsteinii*
GCF_015551715.1	*Parabacteroides goldsteinii*
GCF_015557085.1	*Parabacteroides goldsteinii*
GCF_016117715.1	*Bacteroides thetaiotaomicron*
GCF_016586395.1	*Anaerobutyricum hallii*
GCF_017776255.1	*Anaerobutyricum hallii*
GCF_017776275.1	*Anaerobutyricum hallii*
GCF_017873595.1	*Parabacteroides goldsteinii*
GCF_018140635.1	*Anaerobutyricum hallii*
GCF_018289275.1	*Bacteroides thetaiotaomicron*
GCF_018291825.1	*Bacteroides thetaiotaomicron*
GCF_018784745.1	*Anaerobutyricum hallii*
GCF_019857385.1	*Bacteroides thetaiotaomicron*
GCF_019896115.1	*Bacteroides thetaiotaomicron*
GCF_020091305.1	*Bacteroides thetaiotaomicron*
GCF_020091425.1	*Parabacteroides goldsteinii*
GCF_020557405.1	*Anaerobutyricum hallii*
GCF_022453665.1	*Bacteroides thetaiotaomicron*
GCF_022713225.1	*Prevotella corporis*
GCF_024758405.1	*Bacteroides thetaiotaomicron*
GCF_024760065.1	*Bacteroides thetaiotaomicron*
GCF_024760245.1	*Bacteroides thetaiotaomicron*
GCF_024788545.1	*Parabacteroides goldsteinii*
GCF_024799845.1	*Bacteroides thetaiotaomicron*
GCF_027668695.1	*Anaerobutyricum hallii*
GCF_027688775.1	*Parabacteroides goldsteinii*
GCF_028743775.1	*Prevotella corporis*
GCF_031010215.1	*Prevotella corporis*
GCF_034365545.1	*Parabacteroides goldsteinii*
GCF_036419155.1	*Anaerobutyricum hallii*
GCF_036419675.1	*Bacteroides thetaiotaomicron*
GCF_039535665.1	*Prevotella corporis*
GCF_039733985.1	*Parabacteroides goldsteinii*
GCF_039733995.1	*Parabacteroides goldsteinii*

GCF_040094855.1	*Anaerobutyricum hallii*
GCF_040098145.1	*Parabacteroides goldsteinii*
GCF_041014885.1	*Bacteroides thetaiotaomicron*
GCF_041119805.1	*Parabacteroides goldsteinii*
GCF_041325985.1	*Anaerobutyricum hallii*
GCF_042852775.1	*Parabacteroides goldsteinii*
GCF_900209925.1	*Anaerobutyricum hallii*
GCF_902364815.1	*Anaerobutyricum hallii*
GCF_902375575.1	*Parabacteroides goldsteinii*
GCF_910574435.1	*Parabacteroides goldsteinii*
GCF_910574575.1	*Parabacteroides goldsteinii*
GCF_937930245.1	*Prevotella corporis*
GCF_947253205.1	*Prevotella corporis*

Appendix

**NCBI Accession Number**	***Species Name***
GCF_000159315.1	*Lentilactobacillus hilgardii*
GCF_000182855.2	*Lactobacillus amylovorus*
GCF_000183825.1	*Ligilactobacillus animalis*
GCF_000191545.1	*Lactobacillus amylovorus*
GCF_000194115.1	*Lactobacillus amylovorus*
GCF_000211375.1	*Lentilactobacillus buchneri*
GCF_000298115.2	*Lentilactobacillus buchneri*
GCF_001312845.1	*Secundilactobacillus collinoides*
GCF_001434535.1	*Ligilactobacillus animalis*
GCF_001434655.1	*Lentilactobacillus hilgardii*
GCF_001435215.1	*Lactiplantibacillus argentoratensis*
GCF_001435975.1	*Secundilactobacillus collinoides*
GCF_001623425.1	*Secundilactobacillus collinoides*
GCF_001676805.1	*Levilactobacillus brevis*
GCF_001705475.1	*Ligilactobacillus animalis*
GCF_002117325.1	*Levilactobacillus brevis*
GCF_002117345.1	*Levilactobacillus brevis*
GCF_002706375.1	*Lactobacillus amylovorus*
GCF_002762175.1	*Levilactobacillus brevis*
GCF_002894975.1	*Ligilactobacillus animalis*
GCF_003641165.1	*Lactiplantibacillus argentoratensis*
GCF_004354745.1	*Lentilactobacillus buchneri*
GCF_004354795.1	*Lentilactobacillus hilgardii*
GCF_006228205.1	*Levilactobacillus brevis*
GCF_006228285.1	*Levilactobacillus brevis*
GCF_006384175.1	*Lactobacillus amylovorus*
GCF_007991855.1	*Lactiplantibacillus argentoratensis*

GCF_008369805.1	*Lentilactobacillus buchneri*
GCF_008694025.1	*Lentilactobacillus hilgardii*
GCF_009832765.1	*Lentilactobacillus hilgardii*
GCF_009933595.1	*Ligilactobacillus animalis*
GCF_011765585.1	*Lentilactobacillus hilgardii*
GCF_018314255.1	*Lentilactobacillus buchneri*
GCF_018551565.1	*Lactiplantibacillus argentoratensis*
GCF_018552295.1	*Lactiplantibacillus argentoratensis*
GCF_018552305.1	*Lactiplantibacillus argentoratensis*
GCF_020149995.1	*Lactobacillus amylovorus*
GCF_020295655.1	*Lactiplantibacillus argentoratensis*
GCF_022642685.1	*Lactobacillus amylovorus*
GCF_023523445.1	*Lactobacillus amylovorus*
GCF_025191085.1	*Lentilactobacillus hilgardii*
GCF_025449355.1	*Lactobacillus amylovorus*
GCF_026184355.1	*Lactobacillus amylovorus*
GCF_030062395.1	*Ligilactobacillus animalis*
GCF_030438335.1	*Ligilactobacillus animalis*
GCF_030438385.1	*Ligilactobacillus animalis*
GCF_033096565.1	*Lentilactobacillus buchneri*
GCF_037201785.1	*Levilactobacillus brevis*
GCF_037290055.1	*Lactiplantibacillus argentoratensis*
GCF_039880275.1	*Levilactobacillus brevis*
GCF_044792165.1	*Ligilactobacillus animalis*
GCF_045272285.1	*Lentilactobacillus buchneri*
GCF_048564885.1	*Lentilactobacillus buchneri*
GCF_050410815.1	*Levilactobacillus brevis*
GCF_050847345.1	*Lactiplantibacillus argentoratensis*
GCF_050871075.1	*Lactiplantibacillus argentoratensis*
GCF_051165835.1	*Ligilactobacillus animalis*
GCF_052222745.1	*Lentilactobacillus hilgardii*
GCF_052589095.1	*Lentilactobacillus hilgardii*
GCF_900475625.1	*Levilactobacillus brevis*
GCF_902374015.1	*Lentilactobacillus hilgardii*
GCF_964060345.1	*Lentilactobacillus buchneri*
GCF_964065095.1	*Lentilactobacillus buchneri*
