# Supplementary material for: Bioinformatic Analysis of Oxalate-Degrading Enzymes in Probiotics: A Systematic Genome-Scale and Structural Survey
Source: Microorganisms. 2025 Nov 8;13(11):2553. doi: 10.3390/microorganisms13112553 (PMC12654022; doi:10.3390/microorganisms13112553)
Supplement: Supplementary file 1 [file microorganisms-13-02553-s001.zip › Supplementary Table S3.pdf]

Table S3. Reference protein sequences used for BLAST analysis.

Ref_chain Table	enzyme	PDB_ID
	OXC	2C31
	FRC	1T4C
	OOR	5EXE
	OXDC	1UW8
