# Supplementary material for: Bioinformatic Analysis of Oxalate-Degrading Enzymes in Probiotics: A Systematic Genome-Scale and Structural Survey
Source: Microorganisms. 2025 Nov 8;13(11):2553. doi: 10.3390/microorganisms13112553 (PMC12654022; doi:10.3390/microorganisms13112553)
Supplement: Supplementary file 1 [file microorganisms-13-02553-s001.zip › Supplementary Table S4.pdf]

Table S4. BLAST Alignment-Based Homology Quantification Table with Reference Chain.

Accession Number	Species Name	OXC	FRC(1)	FRC_2
GCF_000022965.1	*B. animalis*	48.60	44.11	—
GCF_034298135.1	*L. acidophilus*	53.26	44.44	—
GCF_000014425.1	*L. gasseri*	50.18	45.83	—
GCF_046109915.1	*L. helveticus*	53.38	NA	36.10
GCF_014058685.1	*L. johnsonii*	49.46	45.60	—
GCF_009184665.1	*L. kefiranofaciens*	51.71	40.93	50.00
GCF_003703885.1	*L. reuteri*	50.18	46.87	—

Accession Number	Species Name	OOR_A	OOR_B	OOR_C
GCF_001689125.2	*Blautia pseudococcoides*	NA	NA	36.81
GCF_025289255.1	*Blautia hydrogenotrophica*	NA	NA	40.86
GCF_041223525.1	*Blautia marasmi*	NA	45.90	NA

Accession Number	Species Name	OXDC
GCF_015553285.1	*Lachnospira_eligens*	34.57
