# Supplementary material for: Bioinformatic Analysis of Oxalate-Degrading Enzymes in Probiotics: A Systematic Genome-Scale and Structural Survey
Source: Microorganisms. 2025 Nov 8;13(11):2553. doi: 10.3390/microorganisms13112553 (PMC12654022; doi:10.3390/microorganisms13112553)
Supplement: Supplementary file 1 [file microorganisms-13-02553-s001.zip › Supplementary Table S5.pdf]

Table S5. BLAST Alignment-Based Homology Quantification Table with *L. gasseri* as Internal Control.

Accession Number	Species Name	OXC	FRC(1)	FRC_2
GCF_000022965.1	*B. animalis*	49.83	70.26	—
GCF_034298135.1	*L. acidophilus*	71.75	84.51	—
GCF_000014425.1	*L. gasseri*	100.00	100.00	—
GCF_046109915.1	*L. helveticus*	75.48	NA	82.16
GCF_014058685.1	*L. johnsonii*	97.06	93.36	—
GCF_009184665.1	*L. kefiranofaciens*	70.97	85.00	86.80
GCF_003703885.1	*L. reuteri*	82.34	81.86	—
