# Supplementary material for: Bioinformatic Analysis of Oxalate-Degrading Enzymes in Probiotics: A Systematic Genome-Scale and Structural Survey
Source: Microorganisms. 2025 Nov 8;13(11):2553. doi: 10.3390/microorganisms13112553 (PMC12654022; doi:10.3390/microorganisms13112553)
Supplement: Supplementary file 1 [file microorganisms-13-02553-s001.zip › Supplementary Table S6.pdf]

Table S6. Sequence homology data of additional probiotic species analyzed in the extended dataset.

Accession Number	Species Name	OXC	FRC
GCF_000182855.2	*L. amylovorus*	51.70	45.14
GCF_000183825.1	*Lig. animalis*	50.00	45.37

Accession Number	Species Name	OXC	FRC
GCF_000183825.1	*L. amylovorus*	71.40	88.24
GCF_000183825.1	*Lig. animalis*	94.29	91.37
